# Association between Variations in Cell Cycle Genes and Idiopathic Pulmonary Fibrosis

**DOI:** 10.1371/journal.pone.0030442

**Published:** 2012-01-23

**Authors:** Nicoline M. Korthagen, Coline H. M. van Moorsel, Nicole P. Barlo, Karin M. Kazemier, Henk J. T. Ruven, Jan C. Grutters

**Affiliations:** 1 Center of Interstitial Lung Diseases, St Antonius Hospital, Nieuwegein, The Netherlands; 2 Division of Heart & Lungs, University Medical Center Utrecht, Utrecht, The Netherlands; 3 Clinical Chemistry, St Antonius Hospital, Nieuwegein, The Netherlands; University Medical Center Freiburg, Germany

## Abstract

Idiopathic pulmonary fibrosis (IPF) is a devastating and progressive lung disease. Its aetiology is thought to involve damage to the epithelium and abnormal repair. Alveolar epithelial cells near areas of remodelling show an increased expression of proapoptotic molecules. Therefore, we investigated the role of genes involved in cell cycle control in IPF. Genotypes for five single nucleotide polymorphisms (SNPs) in the tumour protein 53 (TP53) gene and four SNPs in cyclin-dependent kinase inhibitor 1A (*CDKN1A*), the gene encoding p21, were determined in 77 IPF patients and 353 controls. In peripheral blood mononuclear cells (PBMC) from 16 healthy controls mRNA expression of *TP53* and *CDKN1A* was determined.

Rs12951053 and rs12602273, in *TP53*, were significantly associated with survival in IPF patients. Carriers of a minor allele had a 4-year survival of 22% versus 57% in the non-carrier group (p = 0.006). Rs2395655 and rs733590, in *CDKN1A*, were associated with an increased risk of developing IPF. In addition, the rs2395655 G allele correlated with progression of the disease as it increased the risk of a rapid decline in lung function. Functional experiments showed that rs733590 correlated significantly with *CDKN1A* mRNA expression levels in healthy controls.

This is the first study to show that genetic variations in the cell cycle genes encoding p53 and p21 are associated with IPF disease development and progression. These findings support the idea that cell cycle control plays a role in the pathology of IPF. Variations in *TP53* and *CDKN1A* can impair the response to cell damage and increase the loss of alveolar epithelial cells.

## Introduction

Idiopathic pulmonary fibrosis (IPF) is a severe and relentless lung disease that is characterized by fatal scarring of the lung parenchyma and progressive shortness of breath. IPF is a rare disease with a prevalence of 14 per 100.000 persons [1]. The annual incidence is estimated to be between 4.6 and 7.4 cases per 100.000 persons and about 5 million people are affected worldwide [1]–[3]. Moreover, the incidence continues to rise and IPF is now an important cause of respiratory mortality [3].

The median survival time is only 2.5 to 3.5 years, but individual survival can vary from a few months to >10 years [2], [4]–[6]. Progression of the disease is mainly monitored by lung function testing, and some studies have found that lung function decline is associated with survival time 7,8. However, it is unclear what causes this heterogeneity in survival time and whether it can be predicted by any other means.

The cause of IPF remains unknown but is thought to involve damage to the epithelium and abnormal repair. In 0.5–19% of cases IPF is familial and is most likely caused by a single genetic mutation [9]. Apart from deleterious alleles, no rare risk variants have been found for IPF while a common risk variant in the MUC5B gene has recently been discovered to associate with both familial and sporadic IPF [10]. Susceptibility to IPF and progression of the disease is probably influenced by a combination of genetic variations that drive epithelial injury and abnormal wound healing processes [11].

It has been suggested that IPF pathology has similarities to cancer and that it could be a neoproliferative disease [12]. Previous immunohistological examination of IPF lungs has revealed increased expression of proteins involved in cellular responses to injury and DNA damage, including p53 and p21 [13].

Tumour protein 53 (p53) is a key regulator of apoptosis. It is upregulated upon DNA damage and prevents damaged cells from becoming malignant by inducing growth arrest and cell death [14], [15]. With increasing age, some cells can escape p53-induced cell death and the continued presence of these dysfunctional cells can lead to a decrease in tissue regeneration and repair as well as cancer [16]. Increased levels of p53 in the lungs of IPF patients are consistent with increased apoptosis [13]. Loss of alveolar epithelial cells by apoptosis can impair the regenerative capacity of the lung. P53-induced growth arrest is mediated by increased transcription of cyclin-dependent kinase inhibitor 1A *(CDKN1A)*, the gene encoding p21. The p21 protein (also known as Cip1, Sdi1, and Waf1) regulates cell cycle progression. Induction of this protein prevents proliferation and allows optimal DNA repair thereby reducing apoptosis and cancer risk [17].

Genetic variations may play a role in IPF disease susceptibility and progression and could give important insights into disease aetiology [11], [18].

## Materials and Methods

### Ethics statement

The Ethical Committee of the St. Antonius Hospital approved the study protocol and all subjects gave written informed consent.

### Subjects

77 IPF patients who visited the Centre for Interstitial Lung Diseases at the St. Antonius Hospital, the Netherlands between November 1998 and 2007 were included in this study.

Diagnoses made before 2002 were reviewed by a clinician and patients were only included when the diagnosis met the current ATS/ERS guidelines [ATS/ERS, 2002 565 /id]. Other causes of UIP (drugs, collagen vascular diseases) were ruled out. 58 males and 19 females (mean age 60.8 years {SD 13.6}) were included. In 58 cases the diagnosis of UIP was confirmed on lung biopsy (75%). From 64 patients lung function follow-up was available. In accordance with the method proposed by Egan *et al.* [19], we defined a rapid decline in lung function as more than 15% decline in percent-predicted D_LCO_ (diffusion capacity of the lung for carbon monoxide) or more than 10% decline in percent-predicted vital capacity (VC) over a one-year period. Length of follow-up for survival was up to 4 years and was based on hospital records. Patients that were still alive or transplanted were censored in the survival analysis. Clinical parameters at diagnosis were obtained from hospital records.

The control group consisted of 353 healthy volunteers (mean age 39.2 years {SD 12.4}, 139 males, 210 females). This included 313 self-reported healthy Caucasian employees of the St Antonius hospital and 40 volunteers that underwent brochoalveolar lavage between January and October 2007. The Ethical Committee of the St. Antonius Hospital approved the study protocol (R-05.08A). All subjects that met the inclusion criteria and gave written informed consent were included in this study.

### Genotyping

DNA was extracted from whole blood samples and single nucleotide polymorphism (SNP) typing was conducted using a custom Illumina goldengate bead SNP assay in accordance with the manufacturer's recommendations (Illumina Inc; San Diego, USA). The tagger program [20] was used to select haplotype tagging SNPs (tagSNPs) that represent the genetic variation in a specific region. SNPs were selected using the CEU HapMap panel and covering the gene region plus 2500 basepairs upstream and downstream, based on NCBI build 35. We used an r^2^ threshold for SNPs>0.8 under the pairwise tagging options. Three tagSNPs in the p21 gene, *CDKN1A* located on chromosome 6p21.2, were selected based on a minor allele frequency (MAF) higher than 25% in the Caucasian population and one SNP (rs730506) was added because of its potential regulatory function on protein expression through localization on a transcription factor binding site [21]. For the p53 gene, *TP53* located on chromosome 17p13.1, three tagSNPs were selected by reducing the MAF with 5% increments until three tagSNPs could be selected with a MAF higher than 5%. Two potentially functional SNPs were added (rs16956880 and rs11575997) that could have an effect on splicing [22].

### Messenger RNA levels

We used thawed peripheral blood mononuclear cells (PBMC) from 16 healthy controls. The expression of *CDKN1A* and *TP53* mRNA was analysed by quantitative RT-PCR amplification as described previously [23]. Briefly, total RNA was isolated using an Rneasy microkit (Qiagen, Venlo, the Netherlands) according to the manufacturer's protocol. 0.2 µg RNA was used for first-strand cDNA synthesis with the I-script cDNA synthesis kit (Biorad, Veenendaal, the Netherlands). The obtained cDNA was diluted 1/10 with water of which 4 µl was used for amplification in a reaction volume of 20 µl. The PCR was performed with the RT^2^ Real-Time™ SYBR Green PCR master mix (SA-Biosciences, Frederick, USA) according to the manufacturer's protocol. Samples were amplified using a Biorad MyiQ real time PCR detection system for 40 cycles (10 s at 95°C, 20 s at 61°C and 25 s at 72°C. The copy number of *CDKN1A* was normalized by the housekeeping gene β-actin (*ACTB*).

### Statistics

**Figure 1:**
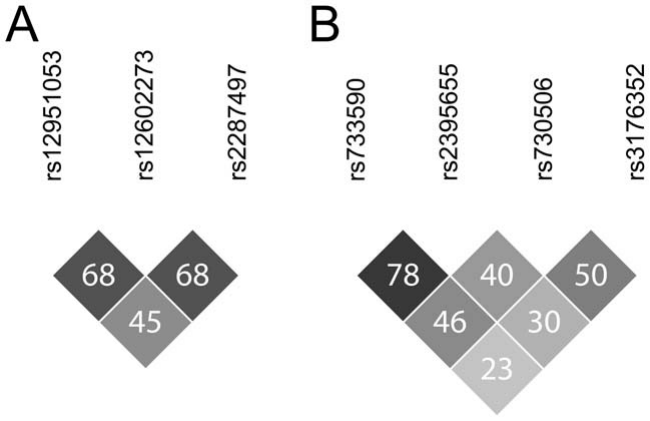
Linkage disequilibrium plot. Showing pairwise r^2^ for SNPs in *TP53* (A) and *CDKN1A* (B).

SPSS 15 (SPSS Inc., Chicago, IL, USA) and Graphpad Prism v. 3 (Graphpad software INC., San Diego, CA, USA) were used for statistical analysis. The Kaplan-Meier method with log-rank test was used to analyse whether any SNPs were associated with survival. Cox regression analysis with covariates was used to check for possible confounders. Pearson's goodness-of–fit Chi-square test and Fisher's exact test were used to test for deviation from Hardy-Weinberg equilibrium and for a difference in genotype and allele frequencies between patients and controls as implemented online at http://ihg2.helmholtz-muenchen.de/cgi-bin/hw/hwa1.pl. Due to linkage disequilibrium between the SNPs (figure 1), the effective number of independent SNPs was 4.46, based on the method proposed by Li and Ji [24] as implemented online at http://gump.qimr.edu.au/general/daleN/matSpD/.

The adjusted significance threshold was set at 0.05/4.46 = 0.011.

## Results

### *TP53*

Genotype and allele frequencies for *TP53* and *CDKN1A* SNPs did not deviate from Hardy-Weinberg equilibrium (table 1).

**Table 1:** *TP53* genotype frequency in patients and controls.

*TP53*	genotype	IPF	controls
rs12951053	AA	0.88 (68)	0.84 (295)
	AC	0.12 (9)	0.16 (56)
	CC	0.0 (0)	0.006 (2)
rs12602273	GG	0.86(66)	0.85 (299)
	CG	0.14 (11)	0.15 (53)
	CC	0.0 (0)	0.003 (1)
rs2287497	GG	0.82 (63)	0.80 (281)
	AG	0.18 (14)	0.19 (68)
	AA	0.0 (0)	0.01 (4)
rs16956880	GG	1.0 (77)	1.0 (353)
rs11575997	CC	1.0 (77)	1.0 (353)

The values in parentheses are the number of individuals the frequency is based on.

There were no significant differences between patients and controls.

There were no significant differences in genotype frequency between patients and controls in *TP53* SNPs. There was linkage disequilibrium between three SNPs (figure 1A): between rs12951053 and rs12602273 D′ = 0.85 and r^2^ = 0.68; between rs12951053 and rs2287497 D′ = 0.78 and r^2^ = 0.45; and between rs12602273 and rs2287497 D′ = 0.98 and r^2^ = 0.68.

**Figure 2:**
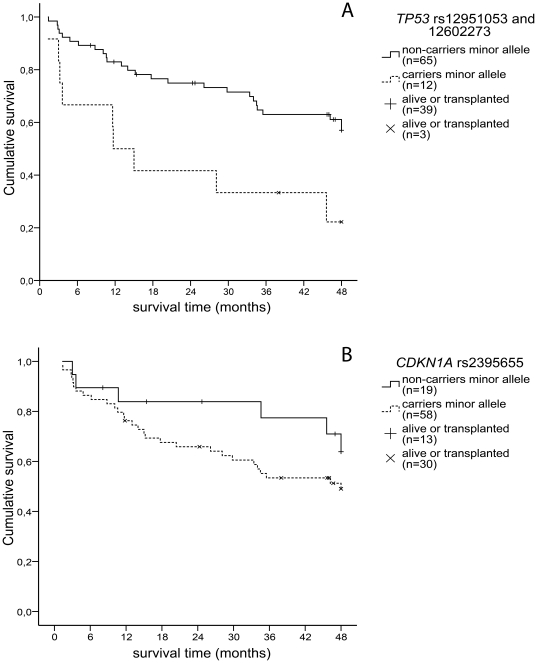
Kaplan-Meier analysis of survival in a cohort of IPF patients. (A) Carriers of the *TP53* rs12951053 C or rs12602273 G alleles had significantly worse 4-year survival rate (p = 0.006). (B) There was no significant difference in survival between carriers and non-carriers of the *CDKN1A* rs2395655 G allele (p = 0.2).

Carriership of the minor alleles of rs12951053 (C) or rs12602273 (G) was significantly associated with shorter survival time, both individually and when the SNPs are combined (figure 2A). Carriers of the minor alleles had a 4-year survival of only 22% versus 57% in the non-carrier group (Kaplan-Meier, Log rank test p = 0.006). Cox regression analysis revealed that age, gender and lung function were no confounding factors. The hazard ratio for carriership of a *TP53* minor allele was 2.9 (95%CI 1.3–6.2, p<0.007). Rs2287497 did not show any association with survival in our IPF cohort.

The two SNPs in *TP53* that were associated with survival were tested for an association with lung function decline (table 2). Carriers of the rs12951053 C allele and carriers of the rs12602273 C allele were more likely to have a rapid decline in lung function, although the difference did not reach statistical significance. In the rs12951053 AC group 5 of 8 (63%) and in the AA group 15 of 50 patients (30%) had a rapid decline in lung function. Together these results suggest that carriership of the *TP53* rs12951053 C allele or rs12602273 C allele predisposes to a rapid progression of IPF.

**Table 2:** Genotype and lung function decline.

	genotype	rapid	Non-rapid
*TP53*			
rs12951053	AA	15	35
	AC	5	3
	CC	0	0
rs12602273	GG	16	34
	CG	4	4
	CC	0	0
*CDKN1A*			
rs733590	TT	3	13
	TC	10	17
	CC	7	8
rs2395655*	AA	1	11
	AG	10	18
	GG	9	9

Number of IPF patients with non-rapid or rapid decline in %predicted vital capacity (>10% in one year) or %predicted diffusion capacity (>15% in one year).

*Fisher's exact test with carriers of p21 rs2395655G vs. non-carriers resulted in p = 0.04.

No differences in *TP53* mRNA expression were observed between the p53 genotypes.

### *CDKN1A*

There was significant linkage disequilibrium between the four *CDKN1A* SNPs (figure 1B): between rs733590 and rs2395655 D′ = 0.96 and r^2^ = 0.78;.between rs733590 and rs730506 D′ = 0.99 and r^2^ = 0.46; between rs733590 and rs3176352 D′ = 0.62 and r^2^ = 0.23; between rs2395655 and rs730506 D′ = 1 and r^2^ = 0.40; between rs2395655 and rs3176352 D′ = 0.76 and r^2^ = 0.30; and between rs730506 and rs3176352 D′ = 0.81 and r^2^ = 0.50.

All four of the SNPs in *CDKN1A* were associated with IPF (table 3). Only the association with rs733590 and rs2395655 remained significant at the adjusted threshold, and the association was strongest for rs2395655. Carriership of rs2395655 *GG* genotype in patients was almost twice as high as in controls (30% versus 16% respectively, p = 0.003). There is a high degree of linkage disequilibrium within the gene but haplotype analysis did not generate superior results (data not shown).

**Table 3:** *CDKN1A* genotype and carriership frequency in patients and controls.

*CDKN1A*	Genotype	IPF	controls	Carriership	IPF	controls	P value
rs733590*	TT	0.31 (24)	0.39 (139)	T	0.74	0.86	CC p = 0.007
	TC	0.43 (33)	0.47 (166)	C	0.70	0.61	OR = 2.2 (1.23–4.03)
	CC	0.26 (20)	0.14 (48)				
rs2395655*	AA	0.25 (19)	0.34 (120)	A	0.70	0.84	GG p = 0.003
	AG	0.45 (35)	0.50 (177)	G	0.76	0.66	OR = 2.3 (1.31–4.05)
	GG	0.30 (23)	0.16 (55)				
rs730506	GG	0.49 (38)	0.59 (208)	G	0.89	0.96	CC p = 0.013
	CG	0.40 (31)	0.37 (132)	C	0.51	0.41	OR = 3.0 (1.21–7.58)
	CC	0.10 (8)	0.04 (13)				
rs3176352	CC	0.39 (30)	0.54 (189)	C	0.87	0.94	CC p = 0.020
	CG	0.48 (37)	0.41 (143)	G	0.62	0.46	OR = 1.8 (1.09–3.00)
	GG	0.13 (10)	0.06 (21)				

The values in parentheses are the number of individuals. P values are based on the number of individuals with and without the specified genotype and are calculated using a Pearson's goodness-of-fit chi-square test. Odds ratio (OR) is shown with the 95% confidence interval in brackets.

*After correction for multiple testing rs733590 and rs2395655 remained significant.

Survival in carriers of rs2395655 G allele was worse than in non-carriers, however, the difference did not reach statistical significance (p = 0.2, figure 2B). Rs2395655 and rs733590 were tested for an association with lung function decline (table 2). The association between rs733590 and lung function decline did not reach statistical significance. Carriers of rs2395655 G allele were more likely to have a rapid decline in lung function (p = 0.04, table 2). Nineteen patients (41%) carrying the G allele had a rapid decline in lung function while only one patient (8%) with the AA genotype had a rapid decline. This did not remain significant after correction for multiple testing.

**Figure 3:**
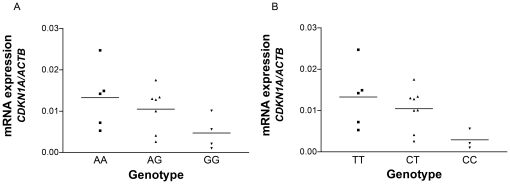
*CDKN1A* mRNA expression in healthy controls. *CDKN1A* mRNA levels were significantly different between rs733590 genotypes, p = 0.03 (TT+CT vs. CC). A similar trend was observed for rs2395655 genotypes (p = 0.06, AA+AG vs. GG).

*CDKN1A* mRNA expression in healthy controls was determined in relation to beta-actin (ACTB) expression and is shown in figure 3. *CDKN1A* mRNA levels were significantly higher in carriers of the rs733590 T allele, using an uncorrected t-test (p = 0.03 TT+CT vs CC). For carriers of the rs2395655 A allele, a similar trend was observed (p = 0.06).

## Discussion

This study reports the novel finding that SNPs in *CDKN1A* predispose to IPF and that SNPs in both *TP53* and *CDKN1A* are associated with progression in IPF. The SNPs in *TP53* had a more pronounced effect on survival while rs2395655 in *CDKN1A* had a more pronounced effect on rapid lung function decline. Survival was significantly correlated with change in lung function in our cohort (p = 8×10^−9^, results not shown) as is usually observed in IPF [25]. Together, our findings show that variations in cell cycle genes are involved in IPF.

Both p53 and p21 are vital cell cycle regulators after DNA damage and are determining factors in cell fate. Inhaled substances, like cigarette smoke, can cause DNA damage to lung cells and exposure to these substances have been associated with an increased risk of developing IPF [26]. In damaged cells, upregulation of p53 occurs and this induces growth arrest and apoptosis. It has been shown that in mice, injury to type II alveolar epithelial cells caused pulmonary fibrosis [27]. Alveolar type II cells are progenitors of type I cells and essential for alveolar repair after induced injury [28]. They react by immediate proliferation along the alveolar basement membrane. Previous studies have detected increased levels of proapoptotic molecules in hyperplastic alveolar epithelial cells in IPF patients [13], [29].

 However, induction of p21 can rescue a cell from apoptosis by allowing DNA repair. In addition, p21 has been reported to be elevated during the differentiation of alveolar epithelial type II cells into type I cells [30]. We found that the *CDKN1A* allele that predisposed to IPF disease development was associated with decreased *CDKN1A* mRNA expression. This finding has to be further investigated in light of previous immunohistochemical findings that showed increased p21 levels in the lungs of IPF patients [13]. The upregulation of p21 in IPF lungs may occur later in disease or may be insufficient to prevent disease progression after injury. Forced expression of the transfected human of *CDKN1A* gene in mice resulted in decreased apoptosis, inflammation and fibrosis after bleomycin installation [31]. In addition, p21 attenuates epithelial mesenchymal transition, a process that contributes to the formation of fibroblast foci [32], and it plays an important role in the prevention of cancer by inducing cell cycle arrest [33]. This dual functionality of p21 may therefore explain both the remodelling in IPF as well as the increased incidence of carcinomas that is thought to occur in IPF patients [34]. Together this indicates that the absence of p21 causes a pro-fibrotic environment, while the induced presence of p21 results in a better healing process.

This study was part of hypothesis generating research and therefore the findings will have to be validated in an independent cohort. The SNPs that were associated with IPF in this study are tagSNPs that represent the genetic variation in the gene region. It is likely that these SNPs are linked to a functional SNP in another part of the gene.

The number of patients included in this study was limited, a problem that almost all studies with IPF patients face. For instance, an association was found between rs2395655 and lung function decline (p = 0.04). However, due to the correction for multiple testing, a cohort of at least 80 patients would be needed to reach the adjusted significance level of p = 0.011 with 80% power. Similarly, at least 46 individuals would be required to reach a power of 80% for the association between rs2395655 and *CDKN1A* mRNA expression. Another limitation of this study is the difference in age between patients and controls. However, no effect of age on genotype distribution was observed.

In conclusion, we found that the *TP53* rs12951053 and rs12602273 SNPs were significantly associated with survival in IPF patients and that *CDKN1A* SNPs rs2395655 and rs733590 were significantly associated with the risk of developing IPF. Furthermore, the *CDKN1A* SNPs were associated with a rapid decline in lung function and significantly decreased *CDKN1A* mRNA levels. This is the first study to show that genetic variation in the genes encoding p53 and p21 might play an important role in IPF. Further studies are needed to elucidate the role of cell cycle genes in IPF pathology.
